# Tick Paralysis: A Thorough Examination May Prevent Unnecessary Harm

**DOI:** 10.7759/cureus.43932

**Published:** 2023-08-22

**Authors:** Farah Salman, Abdallah Atlantawi, Nizar Maraqa

**Affiliations:** 1 General Pediatrics, University of Florida College of Medicine – Jacksonville, Jacksonville, USA; 2 Pediatric Infectious Diseases, University of Florida College of Medicine – Jacksonville, Jacksonville, USA

**Keywords:** dermacentor variabilis, intravenous immunoglobulin, guillain-barré syndrome, ticks, tick paralysis

## Abstract

Tick paralysis is a relatively uncommon tick-borne illness that is often overlooked and misdiagnosed. Therefore, it is not unusual for cases to undergo unnecessary work-up and interventions that may delay correct diagnosis and treatment, placing the patient at risk for catastrophic consequences. We present the case of a four-year-old female who developed ascending flaccid paralysis, initially misdiagnosed with Guillain-Barré syndrome (GBS). She was placed in the pediatric intensive care unit (PICU) for mechanical ventilation after failing to respond to intravenous immunoglobulin (IVIG) administration and plasmapheresis. Later in her hospital course, she was correctly diagnosed to have tick paralysis.

## Introduction

Tick-borne diseases can be categorized into infectious (e.g., Lyme disease) and non-infectious illnesses such as tick paralysis (TP). However, some tick species can transmit infections and cause TP simultaneously, even following short-period body attachment [1-4]. Generally, TP is estimated to be relatively uncommon, and the data about its incidence remain uncertain in the literature due to its rarity and sporadic nature. Nevertheless, mortality can be as high as 12% in undiagnosed cases [1]. TP is a toxin-mediated cause of acute ataxia and ascending flaccid paralysis. It is more prevalent in children, likely due to their smaller body mass allowing the toxins to have a more prominent effect. It occurs more commonly in females, theorized to be due to the increased difficulty of tick detection in thick long hair [1,5]. The diagnosis of TP is commonly overlooked and delayed, leading to unnecessary therapies, interventions, and improper management [1]. Given the spectrum and severity of diseases that can cause acute ataxia with ascending flaccid paralysis, it is especially important to rule out TP in children living in known tick-endemic regions. Ultimately, early diagnosis and intervention are essential to prevent paralysis progression, which might lead to deadly complications like acute respiratory failure.

## Case presentation

A previously healthy four-year-old female presented to our pediatric emergency department (PED) with a chief complaint of rapidly progressing ascending weakness that manifested acutely on the same day of presentation. Accompanying complaints of mild leg pain and gait imbalance prompted evaluation at an outside medical facility where she had a normal head computed tomography (CT) scan. She also had a negative urine toxicology screen (looking for metabolites of benzodiazepines, cannabinoids, amphetamines, opiates, barbiturates, cocaine, and tricyclic antidepressants) to evaluate for possible accidental ingestion leading to drug-induced muscle weakness. Following the initial evaluation, the child was discharged home with a preliminary diagnosis of muscle strain. Shortly after returning home, her weakness progressed to involve the upper extremities, and she began to experience speech difficulties. Therefore, her parents promptly brought her to our PED for further assessment. Further history was not suggestive of recent illnesses or any known sick contacts. There were no historical findings supporting the diagnosis of botulism or myasthenia gravis. The absence of recent travel history to endemic areas led to low suspicion of TP at the PED. The patient was otherwise healthy with no known chronic conditions. She was not receiving any medications and was up to date per the recommended U.S. routine immunization schedule.

Physical examination revealed an alert but anxious child. She was tachypneic with shallow respiration. Her neurological examination was remarkable for bilateral limb hypotonia, significantly decreased muscle strength (distal worse than proximal) in both upper and lower extremities, and markedly diminished deep tendon reflexes. Her pupils were equal, round, and reactive to light. She had weak extraocular movements and occasional dysconjugate gaze with severe bilateral ptosis. She was unable to move her mouth and was crying with a muffled voice. She had a weak to absent gag reflex and weak cough reflex upon suctioning. No sensory deficit or dysautonomia was appreciated. The rest of her recorded examination, including skin and hair, was unremarkable. Brain and spine magnetic resonance imaging (MRI) with contrast was obtained and reported as normal. Cerebrospinal fluid (CSF) analysis, as shown in Table 1, revealed no evidence of acute infection or inflammation and no albuminocytologic dissociation. The presence of red blood cells in the CSF was attributed to the traumatic nature of the lumbar puncture procedure. Given her presentation, the differential diagnoses included Guillain-Barré syndrome (GBS) - with lack of characteristic CSF findings explained by the early course of her illness - and Miller-Fisher variant of GBS given her ataxia, areflexia, and occasional ophthalmoplegia.

**Table 1:** Laboratory results on presentation

Lab Test	Result	Reference Range
CBC with Differential		
White blood cell count	10.18 K/µL (0.01x10^9^/L)	5.50-15.50 K/µL (0.01-0.02x10^9^/L)
Red blood cell count	4.50 mL/µL (4.50x10^12^/L)	3.90-5.30 mL/µL (3.9-5.3x10^12^/L)
Hemoglobin	12.4 g/dL (124 g/L)	11.2-14.3 g/dl (112-143 g/L)
Hematocrit	36.80%	34%-40%
Platelet count	319 K/µL (319x10^9^/L)	150-450 K/µL (150-450x10^9^/L)
Neutrophils %	52%	40%-60%
Lymphocytes %	30%	20%-40%
Monocytes %	7%	2%-8%
Eosinophils %	10%	2%-4%
Basophils %	1%	0%-2%
Routine Chemistry		
Sodium	140 mEq/L (140 mmol/L)	135-145 mEq/L (135-145 mmol/L)
Potassium	4.5 mEq/L (4.5 mmol/L)	3.2-5.1 mEq/L (3.2-5.1 mmol/L)
Chloride	107 mEq/L (107 mmol/L)	98-110 mEq/L (98-110 mmol/L)
Carbon dioxide	21 mEq/L (21 mmol/L)	22-32 mEq/L (22-32 mmol/L)
Glucose level	85 mg/dL (4.72 mmol/L)	60-100 mg/dL (3.3- 5.5 mmol/L)
Blood urea nitrogen	16 mg/dL (5.7 mmol/L)	7-23 mg/dL (2.5- 8.2 µmol/L)
Creatinine	0.23 mg/dL (17.5 µmol/L)	0.40- 1.40 mg/dL (30.5-106 µmol/L)
Anion gap	12 mEq/L (12 mmol/L)	8-16 mEq/L (8-16 mmol/L)
Osmolality calculated	280 mOsm/kg (280 mmol/kg)	280-300 mOsm/kg (280-300 mmol/kg)
Inflammatory Markers		
Erythrocyte sedimentation rate	10 mm/hour	3-13 mm/hour
C-reactive protein	0.3 mg/dL (3 mg/L)	0.5-1.0 mg/dL (5-10 mg/L)
Procalcitonin	0.44 ng/mL	0.00- 0.50 ng/mL
Cerebrospinal Fluid		
Color	Colorless	Colorless
Turbidity	Clear	Clear
RBC count	330/mm^3^	0-10/mm^3^
WBC count	1/mm^3^	0-20/mm^3^
Neutrophils	28%	
Lymphocytes	56%	
Monocytes	26%	
Glucose	49 mg/dL (2.7 mmol/L)	50-75 mg/dL (2.7-4.1 mmol/L)
Protein	24 mg/dL	20-40 mg/dL

The patient was admitted to the pediatric intensive care unit (PICU) for continuous cardiorespiratory monitoring. She received one infusion of intravenous immunoglobulin (IVIG), and gabapentin was added for her neuropathic leg pain. However, her weakness continued to progress, and she ultimately required intubation and mechanical ventilation. Plasmapheresis was recommended by pediatric neurology after the lack of response to IVIG. On the second day of her hospital admission and due to the absence of expected clinical improvement, a thorough physical examination was performed that revealed a tick attached to the posterior aspect of her scalp under a clump of deeply matted hair. The tick was carefully removed and sent to a specialty laboratory for species identification while the child received a second round of plasmapheresis. The tick was later identified to be *Dermacentor  variabilis *(American dog tick). The report also revealed that the identified tick does not transmit *Borrelia burgdorferi*. Following the removal of the tick, the child’s neurological status significantly improved, and she gradually returned to baseline. She was discharged home after seven days of hospitalization to be followed by her pediatrician and to complete a 14-day course of empiric oral doxycycline to cover for a possible tick-borne (e.g., rickettsial) infection.

## Discussion

Acute progressive ascending weakness, as described in this child, can be caused by many serious conditions that require extensive investigations and medical interventions, such as GBS. It can also be caused by TP which can be easily managed by appropriate removal of the attached tick and providing supportive treatment while the patient recovers from the effects of the tick toxins. While eliciting a history of tick exposure - including a detailed inquiry about recent outdoor activities, tick bites, and attached ticks - is crucial for early consideration of TP, the diagnosis should not be excluded in the absence of such history. Careful, thorough examination with special attention to common tick attachment sites like behind the ears, nape of the neck, axillary regions, and skin folds is essential to identify hidden attached ticks. Additionally, TP often involves cranial nerves, resulting in bilateral ptosis, dysconjugate gaze, weak gag reflex, and muffled voice. It should be in the differential diagnosis when considering other conditions that involve multiple cranial nerves (e.g., sarcoidosis or Lyme disease). Conducting a comprehensive cranial nerve examination, and assessing extraocular movements, facial muscles, and gag reflex, aids in identifying these abnormalities. The presence of signs and symptoms suggestive of tick-borne infection, such as fever, rash, or other systemic manifestations is uncommon in cases of TP but may help in making an early diagnosis when present.

**Figure 1:**
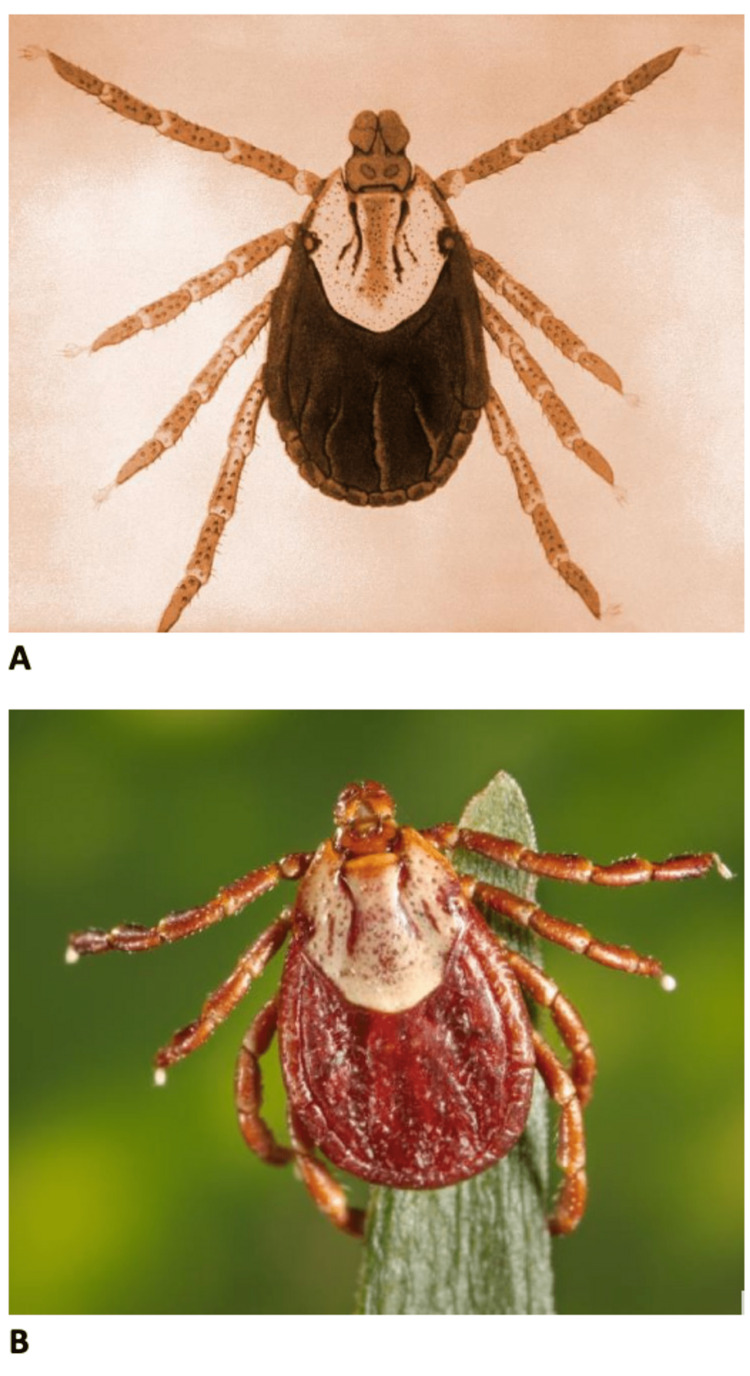
(A) Female American dog tick, Dermacentor variabilis. (B) Female Rocky Mountain Wood tick, Dermacentor andersoni. The images were acquired from the CDC Public Health Image Library (PHIL) are in the public domain and thus are free of any copyright restrictions.

Although many tick species can cause TP, most cases in the United States are caused by two major species: *Deracentor andersoni* (the Rocky Mountain wood tick), which is more prevalent in the western regions, and* D. variabilis* (the American dog tick), more prevalent in the eastern and southern areas (Figures 1A, 1B). In addition to causing TP, these ticks are also well-known vectors for rickettsial disease, such as Rocky Mountain spotted fever (RMSF). Like most other tick-borne illnesses, TP has a peak incidence in the spring and summer. TP occurs more commonly in females and is more prevalent in children [1,5].

Neurotoxins responsible for TP are produced in the gravid female tick’s salivary glands and transmitted during feeding. The highest toxin concentrations are typically delivered during the fifth and seventh day of attachment. The precise mechanism for the development of the symptoms of TP is not fully understood. However, the toxin produced by *Dermacentor* ticks may interrupt sodium flux across axonal membranes in selected locations such as the nodes of Ranvier and nerve terminals. Those toxins produced by the* Ixodes holocyclus* species are thought to inhibit the release of acetylcholine at the presynaptic motor nerve terminal [6,7]. Although there is limited data in the literature to explain the mechanism of neurotoxin spread, the most accepted hypothesis is transported by circulation to motor neurons based on affinity to specific receptors on the membranes of these nerve cells. Several prodromal symptoms (malaise, headache, and vomiting) might precede neurological manifestations. Initially, the patients will start complaining of bilateral lower limb weakness with incoordination and ataxia. If the tick remains in place, the weakness will symmetrically ascend within hours to include the upper body and, ultimately, the respiratory musculature putting the patient in acute severe respiratory failure, coma, and death. Additional symptoms can include paresthesia, muscle pain, irritability, fever, and rash. However, these are usually not endorsed by patients unless suffering from concurrent tick-borne infection (e.g., RMSF or Lyme disease). The sensory exam is typically normal, while the neurologic exam may reveal pupillary dilatation and facial asymmetry secondary to facial musculature involvement [1,6,8].

While GBS is the most prominent condition with overlapping signs and symptoms, other differential diagnoses may be considered like botulism, poliomyelitis, cerebellar ataxia, transverse myelitis, myasthenia gravis, and spinal cord lesion [1,2,9]. The neuroelectrophysiologic findings in TP have been described in the literature and are not pathognomonic. The initial reduction in amplitudes of muscle action potentials evoked by stimulation of motor nerves, prolonged distal motor and sensory latencies, decremental response to 30 Hz stimulation, and reduced conduction velocities was documented. All of these seem to improve after tick removal and resolve with clinical recovery [10,11].

Laboratory testing has no role in the diagnosis, and the first step in establishing the diagnosis of TP is generating a broad differential diagnosis. A thorough physical exam with an emphasis placed on the evaluation of areas where ticks typically attach to their hosts (such as the scalp, axilla, interdigital spaces, and perineum) is needed to expediently detect and remove a symptom-causing tick. Complete removal of the tick, including mouthparts with their salivary glands, is essential for the recovery since the salivary glands are the source of the neurotoxin. The tick should be picked, using a fine tweezer, as close to the skin as possible. Extra care should be taken to apply a gentle motion to pull the tick up without squeezing or twisting its body to ensure that the toxin-containing mouthparts remain intact. It takes about 24-48 hours for a complete recovery after the successful removal of Dermacentor ticks, although weakness and paralysis can occasionally worsen during this time period [12,13]. Thus, patients with paralysis or weakness due to *I. holocyclus *ticks should be observed carefully following tick removal [7]. To minimize tick bites in general, the CDC suggests implementing several preventive measures [14]. It recommends wearing protective clothing when spending time outdoors in areas where ticks are common, using an EPA-registered insect repellent, treating clothing with products containing 0.5% permethrin, checking for ticks after spending time outdoors, and closely inspecting areas such as the scalp, nape of the neck, armpits, groin and behind the ears, removing ticks properly, and showering soon after returning from outside.

## Conclusions

Although TP at its worst can lead to morbidity and death, when diagnosed early it can be a relatively, low-cost and preventable condition. Due to its rarity, the challenging task of locating and identifying the attached tick, and the similarity of presentation with GBS, TP is frequently misdiagnosed. Unfortunately, this may delay the accurate diagnosis and lead to unnecessary interventions and treatments allowing the paralysis to progress with serious complications. Providers should not exclude the possibility of TP due to the absence of tick-exposure history but rather conduct a very thorough examination focusing on areas of likely tick attachment, especially in children.
